# Fast reconstruction of SMS bSSFP myocardial perfusion images using noise map estimation network (NoiseMapNet): a head-to-head comparison with parallel imaging and iterative reconstruction

**DOI:** 10.3389/fcvm.2024.1350345

**Published:** 2024-07-11

**Authors:** Naledi Lenah Adam, Grzegorz Kowalik, Andrew Tyler, Ronald Mooiweer, Alexander Paul Neofytou, Sarah McElroy, Karl Kunze, Peter Speier, Daniel Stäb, Radhouene Neji, Muhummad Sohaib Nazir, Reza Razavi, Amedeo Chiribiri, Sébastien Roujol

**Affiliations:** ^1^ School of Biomedical Engineering and Imaging Sciences, Faculty of Life Sciences and Medicine, King’s College London, London, United Kingdom; ^2^ MR Research Collaborations, Siemens Healthcare Limited, Camberley, United Kingdom; ^3^ Cardiovascular Predevelopment, Siemens Healthcare GmbH, Erlangen, Germany; ^4^ MR Research Collaborations, Siemens Healthcare Limited, Melbourne, VIC, Australia; ^5^ Royal Brompton Hospital, Guy’s and St Thomas NHS Foundation Trust, London, United Kingdom

**Keywords:** magnetic resonance imaging, myocardial perfusion, simultaneous multi-slice, image reconstruction, deep learning

## Abstract

**Background:**

Simultaneous multi-slice (SMS) bSSFP imaging enables stress myocardial perfusion imaging with high spatial resolution and increased spatial coverage. Standard parallel imaging techniques (e.g., TGRAPPA) can be used for image reconstruction but result in high noise level. Alternatively, iterative reconstruction techniques based on temporal regularization (ITER) improve image quality but are associated with reduced temporal signal fidelity and long computation time limiting their online use. The aim is to develop an image reconstruction technique for SMS-bSSFP myocardial perfusion imaging combining parallel imaging and image-based denoising using a novel noise map estimation network (NoiseMapNet), which preserves both sharpness and temporal signal profiles and that has low computational cost.

**Methods:**

The proposed reconstruction of SMS images consists of a standard temporal parallel imaging reconstruction (TGRAPPA) with motion correction (MOCO) followed by image denoising using NoiseMapNet. NoiseMapNet is a deep learning network based on a 2D Unet architecture and aims to predict a noise map from an input noisy image, which is then subtracted from the noisy image to generate the denoised image. This approach was evaluated in 17 patients who underwent stress perfusion imaging using a SMS-bSSFP sequence. Images were reconstructed with (a) TGRAPPA with MOCO (thereafter referred to as TGRAPPA), (b) iterative reconstruction with integrated motion compensation (ITER), and (c) proposed NoiseMapNet-based reconstruction. Normalized mean squared error (NMSE) with respect to TGRAPPA, myocardial sharpness, image quality, perceived SNR (pSNR), and number of diagnostic segments were evaluated.

**Results:**

NMSE of NoiseMapNet was lower than using ITER for both myocardium (0.045 ± 0.021 vs. 0.172 ± 0.041, *p* < 0.001) and left ventricular blood pool (0.025 ± 0.014 vs. 0.069 ± 0.020, *p* < 0.001). There were no significant differences between all methods for myocardial sharpness (*p* = 0.77) and number of diagnostic segments (*p* = 0.36). ITER led to higher image quality than NoiseMapNet/TGRAPPA (2.7 ± 0.4 vs. 1.8 ± 0.4/1.3 ± 0.6, *p* < 0.001) and higher pSNR than NoiseMapNet/TGRAPPA (3.0 ± 0.0 vs. 2.0 ± 0.0/1.3 ± 0.6, *p* < 0.001). Importantly, NoiseMapNet yielded higher pSNR (*p* < 0.001) and image quality (*p* < 0.008) than TGRAPPA. Computation time of NoiseMapNet was only 20s for one entire dataset.

**Conclusion:**

NoiseMapNet-based reconstruction enables fast SMS image reconstruction for stress myocardial perfusion imaging while preserving sharpness and temporal signal profiles.

## 1 Introduction

First-pass cardiac magnetic resonance (CMR) perfusion imaging is recommended by international guidelines for the assessment of patients with known or suspected coronary artery diseases (CAD) (1, 2). The standard and clinically recommended cardiac perfusion MRI sequence uses an electrocardiogram (ECG) triggered, saturation recovery, dynamic contrast enhanced acquisition of at least 3 short axis slices with in-spatial resolution of 2–3 mm performed under stress conditions (3). Hybrid echo planar imaging, fast low angle shot (FLASH), and balanced Steady State Free Precession (bSSFP) readouts have been employed in this context (4). Of these, bSSFP offers an intrinsically higher SNR, and contrast to noise ratio, making it desirable for this application (4).

Improving the spatial coverage and resolution of CMR perfusion imaging has the potential to result in higher diagnostic confidence and accuracy. Higher spatial coverage may facilitate sampling of all myocardial perfusion territories. It could also improve the quantification of total ischemic burden which has been shown to have high prognostic value in studies based on inherently three-dimensional nuclear imaging techniques (5). Higher in-plane spatial resolution, is also desirable as it reduces dark rim artifacts (6), a confounding factor for perfusion defects, and improves quantification of transmural perfusion gradients, a strong predictor of haemodynamically significant CAD (7, 8). Three dimensional (3D) MR perfusion imaging techniques offer increased spatial coverage, but commonly have prolonged acquisition times leading to increased susceptibility to breathing motion (9) and limited in-plane spatial resolution (10). Furthermore, 2D high resolution images were shown to be more sensitive than 3D images with lower resolution to ischemia (11).

Simultaneous multi-slice (SMS) imaging is an alternative acceleration technique which provides increased spatial coverage in the slice direction without compromising in-plane spatial resolution (12). SMS uses multiband radiofrequency pulses to excite multiple separate anatomical slices at the same time, which are then acquired using a shared read-out. To facilitate their separation during the reconstruction process, the simultaneously excited slices are shifted with respect to each other in the field of view which can be achieved by means of Controlled Aliasing in Parallel Imaging Results in Higher Acceleration (CAIPIRINHA) encoding (12). The application of SMS with a FLASH readout has been successfully demonstrated for CMR perfusion (13–16). The combination of CAIPIRINHA and bSSFP is more complex as bSSFP conformant phase cycling and magnetization steady state need to be maintained. This was addressed using a modified RF phase cycling scheme (17) and the gradient-controlled local Larmor adjustment (GC-LOLA) approach (18). This SMS bSSFP approach was successfully demonstrated for CMR perfusion (19–22) which enabled high spatial resolution, increased spatial coverage, improved image quality, and high diagnostic value (21, 23).

SMS images can be reconstructed using standard parallel imaging approaches such as adapted SENSE, SMASH and GRAPPA algorithms (12). However, such reconstructions result in a significant noise enhancement at higher acceleration factors required to achieve high spatial resolution (14). Alternatively, iterative reconstruction techniques using regularization terms can be employed to reconstruct higher SNR images and improve image quality (21). However, iterative techniques are often associated with signal infidelity due to the employed regularization terms and can sometimes introduce some spatio-temporal artifacts (14, 21, 24). Furthermore, iterative reconstructions can be computationally intensive, limiting their integration into clinical routine.

Deep learning-based reconstructions have been proposed to accelerate iterative reconstruction techniques by mapping aliased SMS images to reconstructed images (25, 26). However, these networks were trained using images reconstructed from iterative reconstructions thus limiting their performance. A physics-guided, signal intensity informed and dependent deep learning approach was recently proposed and provided improved image quality over the aforementioned techniques in a small-cohort study of four healthy subjects (24). Signal discrepancies with respect to standard parallel imaging reconstruction were reduced, although not eliminated. The performance of this technique in patients and during stress CMR perfusion where motion is more pronounced has not been evaluated.

Alternatively, image-denoising algorithms may represent a suitable avenue to address the noise enhancement of SMS perfusion images reconstructed using standard parallel imaging reconstruction techniques. A variety of techniques has been proposed for image denoising in the field of computer vision (27, 28). Traditional techniques that do not involve deep learning were successfully employed for image denoising but were often associated with additional blurring artifacts, loss of effective spatial resolution and long computation time. Recently, deep learning techniques have been proposed for image denoising, including in the context of MRI, which have low computational cost upon deployment. Different network architectures have been investigated including the convolutional neural network (CNN) (27) and U-Net architectures. In these techniques, the measured noisy image is directly mapped to a denoised image. Alternatively, a deep learning network can be trained to map a noisy image to its corresponding predicted noise map, which is then subtracted from the original noisy image to generate the denoised image. This reduces the network dependence on image features, potentially improving its generalization and minimizing loss of image sharpness (29). In this study, a U-Net inspired by the denoising CNN (DnCNN) (30) was chosen for the denoising task. Previous studies have shown that the U-Net can offer better results at high noise levels compared to the DnCNN whilst offering a highly reduced training time (31). The potential of deep learning-based image denoising of SMS perfusion images has not specifically been studied.

In this study, a noise map estimation network (NoiseMapNet) was developed for fast, sharpness-preserving, noise-filtered reconstruction of SMS-bSSFP myocardial perfusion images. The proposed reconstruction consists of standard parallel imaging reconstruction followed by non-rigid registration, and deep learning-based image denoising using NoiseMapNet. This technique was retrospectively evaluated in 17 patients who underwent stress CMR perfusion using SMS-bSSFP and was compared to (1) a standard temporal parallel imaging reconstruction based on TGRAPPA with retrospective motion compensation and (2) an iterative reconstruction approach with integrated motion compensation (ITER). A subset of the data presented in this manuscript was presented at the 2023 ISMRM conference.

## 2 Methods

The study was approved by the National Research Ethics Service (15/NS/0030), and written informed consent was obtained from all patients. All imaging was performed on a 1.5 T MRI scanner (MAGNETOM Aera, Siemens Healthcare, Erlangen, Germany) using a 32-element spine array RF coil and an 18-element body array coil during the acquisition.

### 2.1 Proposed myocardial perfusion MRI approach

Dynamic contrast enhanced CMR perfusion measurements were based on an ECG-triggered, saturation recovery, SMS-bSSFP research sequence (21). SMS-bSSFP was implemented with a multiband factor of 2 using RF phase based CAIPIRINHA encoding as proposed by Stäb et al., where the first and second SMS slices were subject to a k-space phase modulation of π/2 and 3π/2, respectively (17). This phase cycling scheme results in a half-FOV shift between the two SMS slices in the phase encoding direction. It also generates a slice-specific shift of the frequency response, which was corrected for using GC-LOLA (18). A lean SMS implementation as previously described (18) was employed and k-space undersampling and image reconstruction were carried out using the system's built-in TGRAPPA reconstruction framework using sum of squares for multicoil data combination. This lean implementation facilitates a one-dimensional joint un-aliasing of simultaneously excited slices and additional in-plane undersampling artifacts and reconstructs the simultaneously excited slices next to each other into an increased/oversampled phase FOV.

The TGRAPPA reconstruction was followed by inline non-rigid image registration (commercially available from the manufacturer) and deep learning-based image denoising. TGRAPPA and non-rigid image registration used the standard inline implementation provided by the vendor. Deep learning-based image denoising was performed offline using a proposed 2D noise map estimating U-Net (NoiseMapNet), as described in the next section.

### 2.2 NoiseMapNet

**Figure 1:**
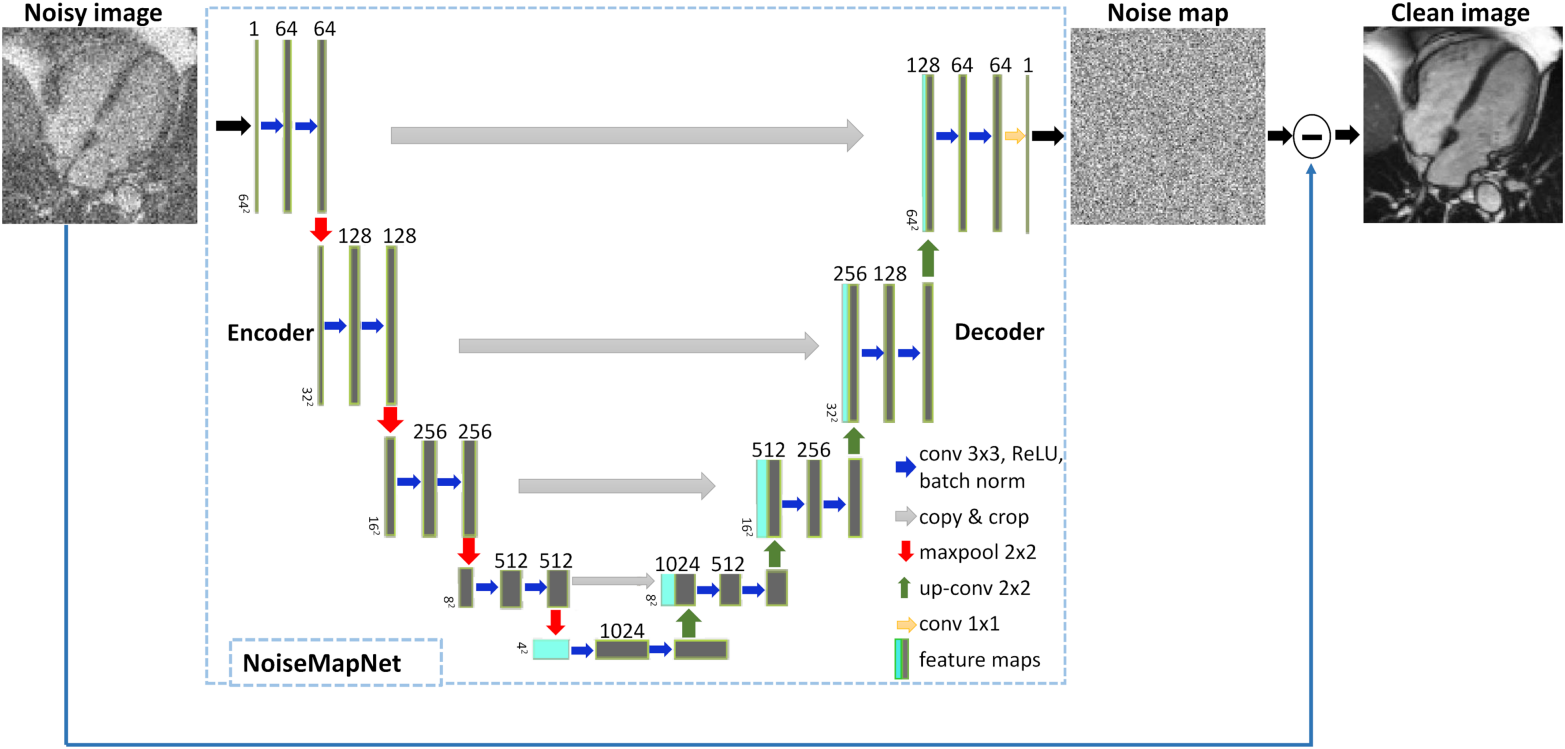
Proposed image denoising approach using NoiseMapNet. NoiseMapNet takes a noisy image as input and outputs a predicted noise map, which is then subtracted from the input image to generate a denoised image.

Figure 1 shows the proposed 2D NoiseMapNet developed for image denoising. NoiseMapNet is based on the U-Net architecture (32) from Medical Open Network for AI (33), taking as an input a magnitude image and outputting its predicted corresponding noise map. The denoised image is generated by subtraction of the predicted noise map from the input noisy image.

As demonstrated in Figure 1, the network is symmetrical, consisting of an encoder and decoder paths with skip connections to copy and propagate high frequency details between the paths. The encoder has a depth of 4 and 64 initial convolutional filters (size 3 × 3, stride = 1) as it has been shown to result in a good-performing U-Net (32). The filters are followed by a rectified linear unit activation function (ReLU), batch normalization to reduce overfitting and improve performance, and a downsampling 2 × 2 maximum pooling (stride 2). The decoder performs upsampling using deconvolutions and outputs the predicted noise map.

### 2.3 Training data

Since it is difficult to generate noise-free images, training was based on high SNR cine images (SNR of 113 ± 32 in the left ventricular blood pool) acquired at different cardiac phases which were considered as an approximation of noise-free data. Four-chamber single-slice cine images acquired from 50 patients referred for clinical CMR were used during the training. All cine images were acquired using a standard cine bSSFP sequence and the following parameters: TR/TE/*α* = 2.3 ms/1.16 ms/53°, FOV = 300 × 250 mm^2^, GRAPPA acceleration factor 2, in-plane resolution = 1.2 × 1.2 mm^2^, and slice thickness = 6 mm. All cardiac phases were used for the training. Gaussian noise was added to the magnitude cine images to simulate an SNR of ∼10 in the left ventricular (LV) blood pool. Image pre-processing steps were resizing using zero-padding for a consistent image size across all datasets, patch selection, and standardization for image scaling. Overall, 1,298 cine images were used for the training procedure and were split into training (80%) and validation (20%) sets with no data leak between the two sets ensured by separating the patient datasets into training and validation datasets.

The impact of image orientation, spatial resolution and simulated SNR on the training and denoising performance of NoiseMapNet is presented in Supplementary Material.

### 2.4 Training procedure

Training of NoiseMapNet was performed by minimizing the standard L2 loss (34) between the predicted noise map and ground truth noise map (30). The ADAM optimizer was used with *β*_1 _= 0.9, *β*_2 _= 0.99 (26) and a learning rate initially optimized to 1 × 10^−5^. A dropout with rate of 0.5 was employed to reduce overfitting. Patch-based learning was employed for its potential to relatively faster training (35, 36) and improved handling of spatially varying noise levels (37). A batch size of 1 and a patch size of 64 × 64 were used. Note that the simulated noise was generated independently for each patch and at each epoch.

Training was conducted for 500 epochs. The model was trained to convergence and early stopping was implemented. Early-stopping was achieved by monitoring the validation loss at the end of every epoch and training was stopped when no improvement was observed in the past 20 epochs. Implementation of the network training was done with the Pytorch framework using Python (version 3.10.7) on a Linux (Ubuntu 16.04) computer with a GPU (Titan V, NVIDIA). Training of the model required approximately 4 h.

### 2.5 In vivo evaluation

Seventeen patients (12 males and 5 females with mean age of 60 ± 11 years) with suspected coronary artery disease and referred for stress cardiac MRI were retrospectively recruited for this study. None of these patients were part of the cohort used for the training of NoiseMapNet. Stress was induced pharmacologically using intravenous adenosine administration at 140 µg/kg/min for at least 3 min. At peak stress, 0.075 mmol/kg of Gadobutrol (Gadovist, Bayer) followed by 25 ml of normal saline flush were injected using a power injector. First-pass CMR perfusion was performed using a dynamic, ECG-triggered, saturation recovery SMS bSSFP research sequence described above. Images were acquired in the short-axis orientation with the following imaging parameters: TE/TR = 1.24 ms/1.9 ms, flip angle = 50°, field of view (FOV) = 360 × 360 mm^2^, 6 slices, resolution = 1.9 × 1.9 mm^2^, slice thickness = 10 mm, 80 dynamics, saturation time = 94 s, bandwidth = 1,302 Hz/px, in-plane acceleration factor = 3.5, multiband factor = 2 (overall acceleration factor = 7). A 32-channel spine coil and an 18-channel body array coil were used. All data were acquired under breath-hold conditions, initiated immediately before contrast arrival in the left chamber. Images were reconstructed with: (a) standard reconstruction using TGRAPPA followed by non-rigid image registration (thereafter referred to as TGRAPPA), (b) iterative reconstruction with integrated motion correction and temporal regularization (ITER) (38), and (c) the proposed NoiseMapNet-based reconstruction [which corresponds to reconstruction (a) followed by NoiseMapNet-based image denoising].

Briefly, ITER combines a preliminary non-rigid motion estimation step with an iterative reconstruction using temporal regularization, which was successfully validated for the reconstruction of SMS bSSFP perfusion images (38). For motion estimation, data are first resampled to a lower resolution, reconstructed using a Conjugate-Gradient SENSE algorithm without any temporal constraint to preserve motion fidelity, and undergo histogram equalization to limit the impact of high intensity signals. Non-rigid motion fields are then estimated and integrated in the final reconstruction as part of the temporal regularization. Further details of ITER can be found in (38).

### 2.6 Quantitative analysis

Myocardial sharpness was quantified at the septal blood-myocardium boundary in the dynamic image corresponding to maximum signal enhancement in the LV blood pool. The myocardial sharpness index was calculated from a single mid-ventricular slice as previously described (20, 39). Briefly, a curve was manually drawn on both sides of the septal endocardium from which tightly spaced profiles across the myocardium-blood interface were generated. A sharpness index was calculated for each profile as 1/d, where d represents the distance over which the signal intensity increases from 20% to 80% of the signal range. An average sharpness index is finally calculated over all profiles for increased robustness.

Additionally, the signal intensity profiles measured with NoiseMapNet and ITER were compared to reference TGRAPPA profiles. The normalized mean squared error (NMSE) was computed across all patients for NoiseMapNet and ITER during the first-pass for both the myocardium (septum) and LV blood pool in one mid-ventricular slice. Since the signal intensities from ITER and TGRAPPA had different scaling, ITER was first scaled to TGRAPPA using the scaling factor minimizing NMSE of ITER (over the first-pass) for each profile. No scaling between TGRAPPA and NoiseMapNet was performed to demonstrate that NoiseMapNet does not introduce any bias.

### 2.7 Qualitative analysis

Subjective assessment of image quality, perceived SNR and number of diagnostic segments was performed by consensus of two expert clinicians (AC and MSN, with over 15 and 7 years' experience respectively) blinded from the patient information and reconstruction technique. The images were visualized with Radiant image viewer. Image quality was scored using a 4-point scale (0 = poor, 1 = major artifact, 2 = minor artifact, 3 = excellent). Perceived SNR (pSNR) was scored using also a 4-point scale (0 = very poor, 1 = major noise, 2 = minor noise, 3 = high SNR). Lastly, the number of diagnostic myocardial segments, as defined by the 16-American Heart Association segments mode (40), was evaluated.

### 2.8 Statistical analysis

Statistical analysis was performed using MATLAB version R2022b (The MathWorks, Natick, MA). Kruskal-Wallis test and one-way ANOVA test were used to compare the three reconstruction techniques in terms of qualitative metrics (image quality, perceived SNR, and number of diagnostic segments) and quantitative metrics (myocardial sharpness), respectively. Statistical significance was defined as a *p*-value <0.05. When Kruskal-Wallis test and One-way ANOVA test demonstrated statistical significance, Wilcoxon signed rank test and paired *t*-test were applied, respectively, using Bonferroni correction, which resulted in a statistical significance threshold of 0.05/3 = 0.017. NMSE obtained using ITER and NoiseMapNet were compared using a paired *t*-test, with statistical significance defined as a *p*-value <0.05.

## 3 Results

**Figure 2:**
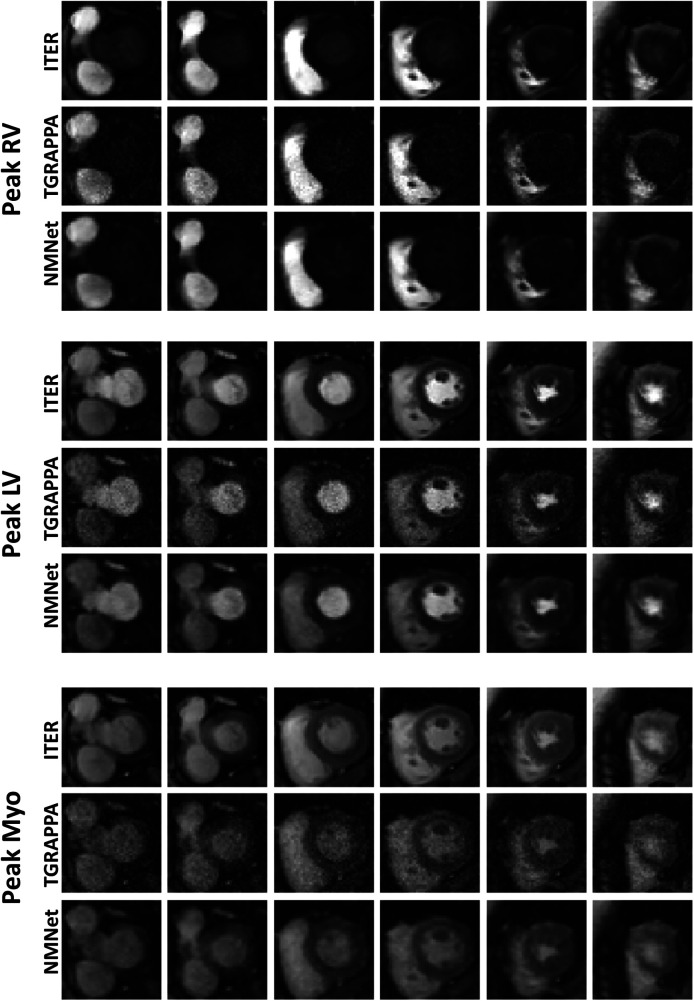
SMS perfusion images acquired in a 67 years old female patient. Images were reconstructed using ITER, TGRAPPA and NoiseMapNet. While high noise level can be observed in TGRAPPA images, image quality and SNR visually improved using the NoiseMapNet.

**Figure 3:**
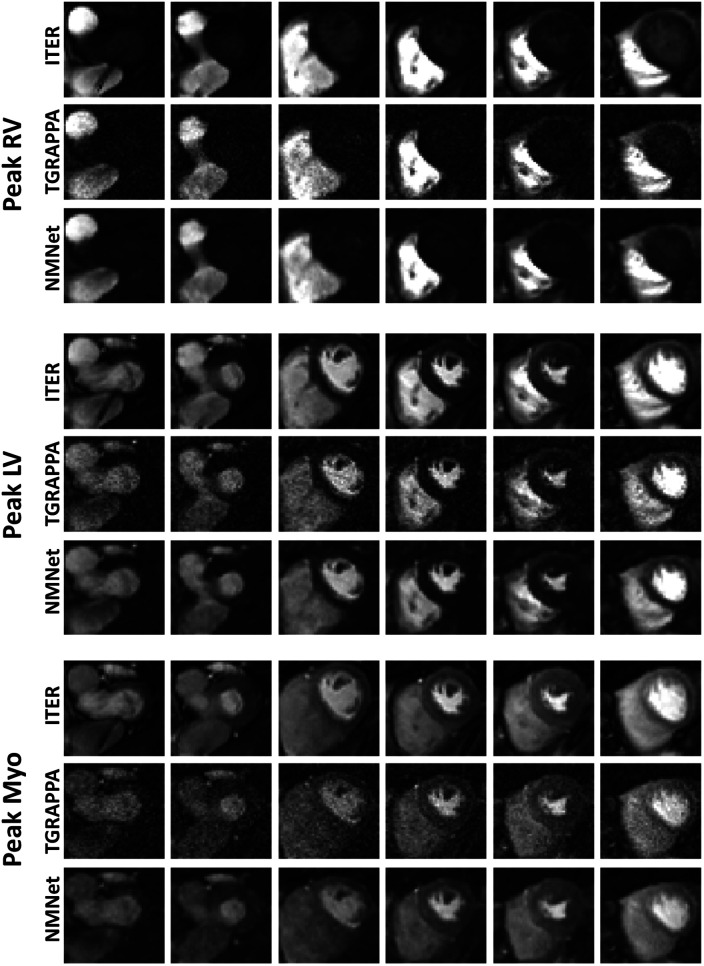
SMS perfusion images acquired in a 44 years old male patient. Images were reconstructed using ITER, TGRAPPA and NoiseMapNet. While high noise level can be observed in TGRAPPA images, image quality and SNR visually improved using the NoiseMapNet. Note the presence of artifacts using ITER (see the most basal slices) which are minimal in both TGRAPPA and NoiseMapNet.

Images from two patients, reconstructed with the three approaches, are shown in Figures 2, 3. Videos showing the entire first-pass of the contrast bolus, for both patients and all reconstruction techniques, are included as Supporting Information (Supplementary Videos S1, S2). A high noise level can be observed in the standard TGRAPPA reconstruction which was substantially lower in images reconstructed using NoiseMapNet and ITER.

**Figure 4:**
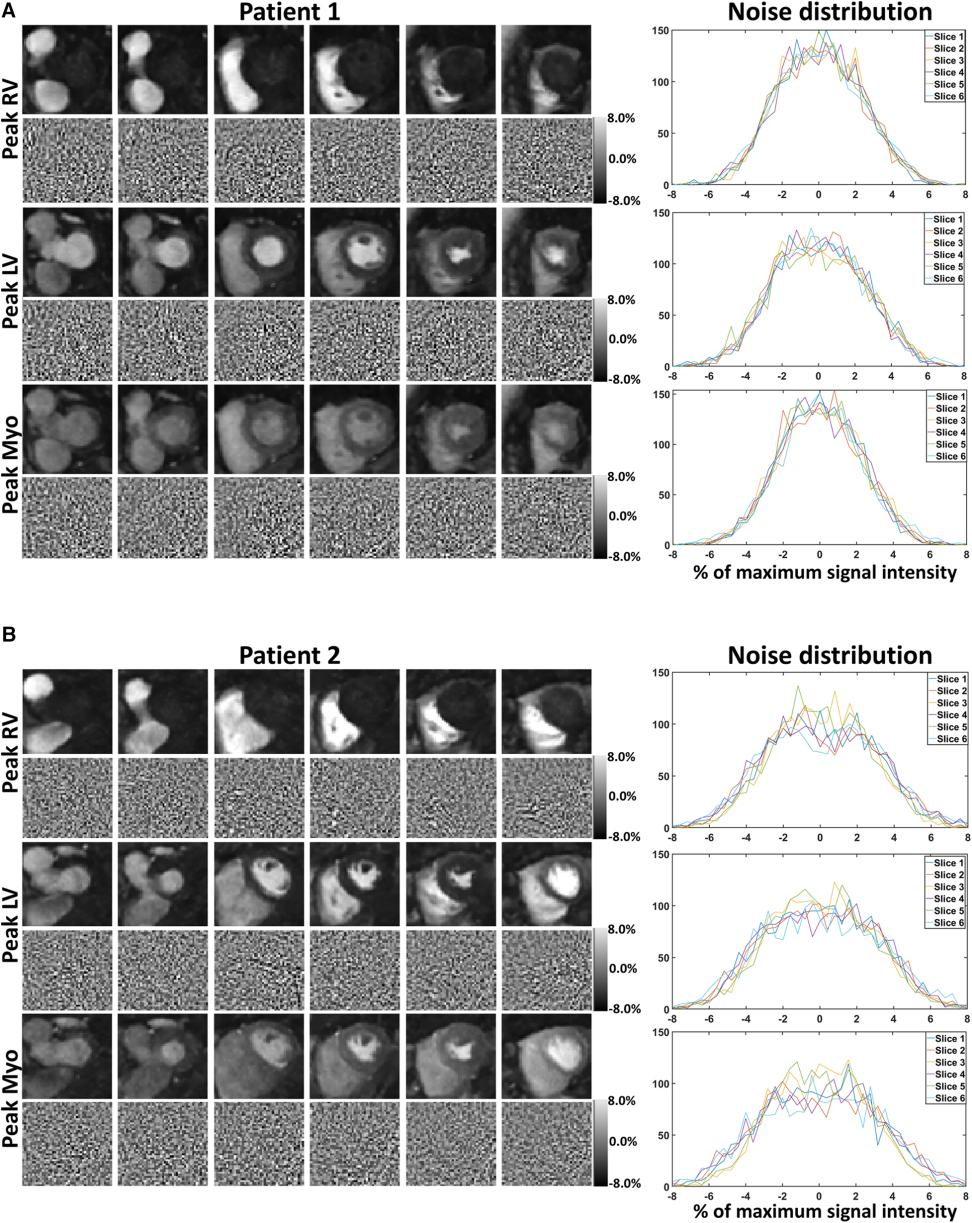
Noisemapnet reconstructed images, corresponding estimated noise maps, and noise distributions in the same example patients and anatomical areas as Figures 2, 3. Minimal anatomical structures are visible in the noise maps, suggesting that the proposed method conserves image sharpness and features in the denoised images. Noise distribution plots follow a Gaussian distribution as expected from the model.

**Figure 5:**
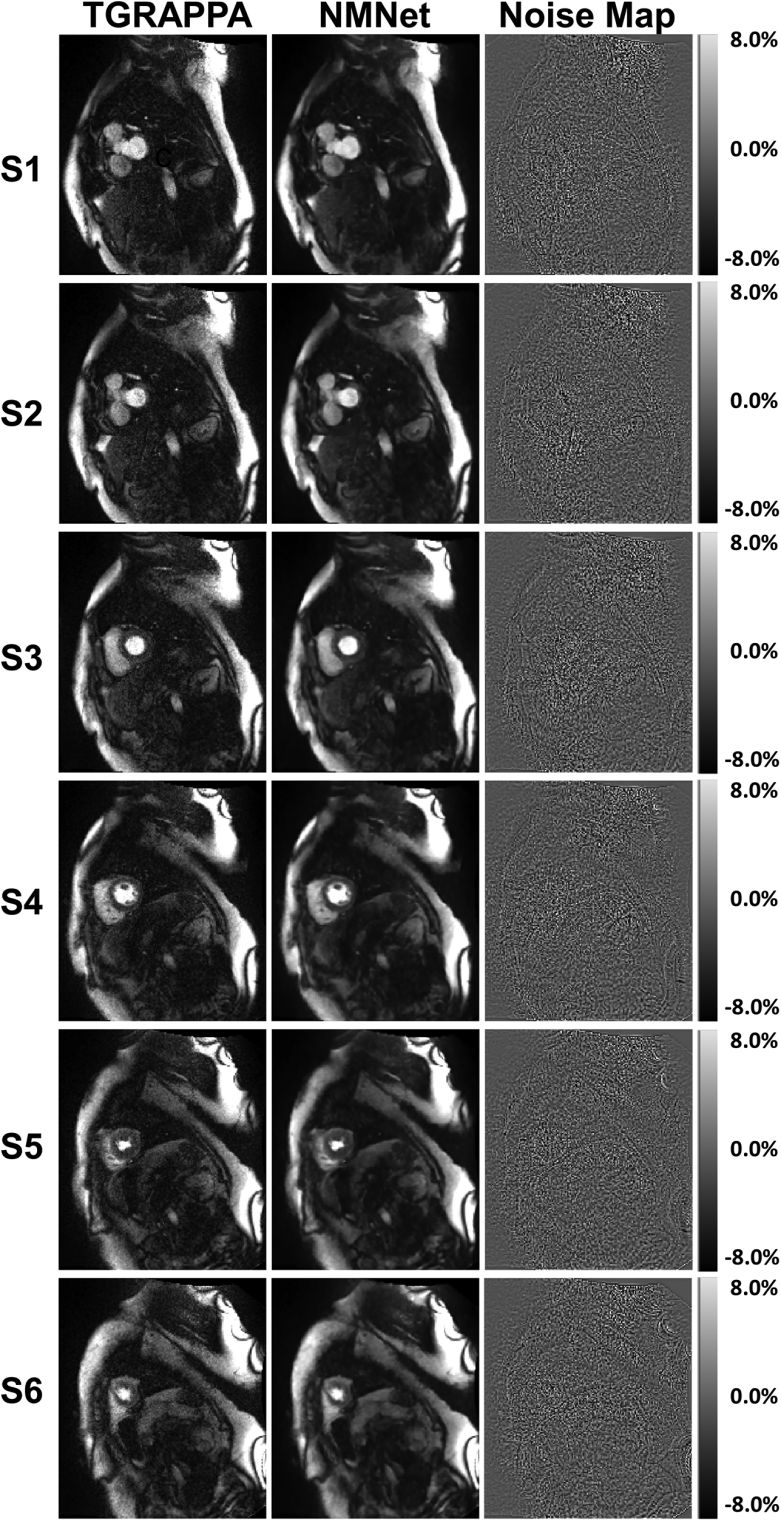
TGRAPPA images, NoiseMapNet (NMNet) images and predicted noise maps are shown for the patient shown in Figure 2 and all 6 SMS slices acquired at maximum signal enhancement in the left ventricular blood pool. Significant spatial noise variations are observed in the noise maps. NoiseMapNet provided consistent image denoising across the entire field of view, despite spatially varying noise levels.

Figure 3 and Supplementary Video S2 also show the presence of artifacts using ITER (see the most basal slices) which are minimal in both TGRAPPA and NoiseMapNet. The corresponding noise maps estimated with NoiseMapNet from the same two patients are shown in Figure 4 and in the Supplementary Videos S3, S4 for all dynamic frames. Minimal anatomical structures are visible in the noise maps indicating the ability of NoiseMapNet to conserve image sharpness and features in the denoised images. Figure 5 illustrates the performance of NoiseMapNet across the entire field of view in the patient from Figure 2. NoiseMapNet provided consistent image denoising across the entire field of view, despite spatially varying noise levels, as previously observed with other patch-based learning approach (37).

**Figure 6:**
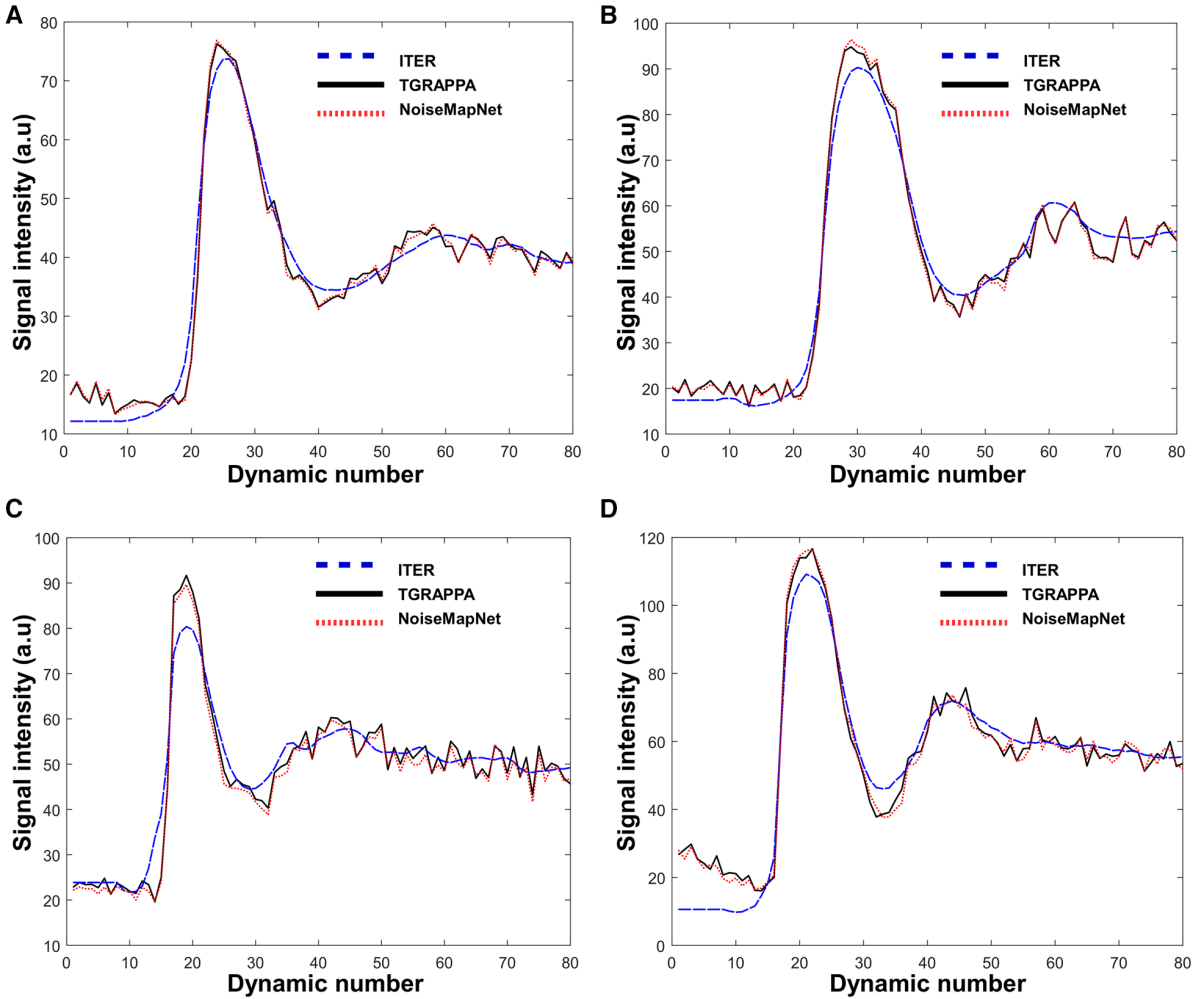
Signal intensity profiles measured in the LV blood pool over all dynamics in four patients. ITER resulted in some alteration of the temporal signal profile with respect to reference TGRAPPA. The temporal profiles measured with NoiseMapNet and TGRAPPA were visually consistent.

Figure 6 shows the temporal signal profiles measured in the LV blood pool for each reconstruction from four patients. ITER resulted in some alterations of the temporal profiles of the signal with respect to the reference TGRAPPA signal profiles. Specifically, temporal smoothing can be observed in ITER as earlier contrast uptake and delayed/reduced signal peak as expected from the temporal regularization employed in this reconstruction. Conversely, NoiseMapNet and TGRAPPA signal profiles appears similar, suggesting the absence of bias when using NoiseMapNet. This was confirmed over all patients where the NMSE over the first-pass of NoiseMapNet was lower compared to that of ITER for both myocardium (0.045 ± 0.021 vs. 0.172 ± 0.041, *p* < 0.001) and left ventricular blood pool (0.025 ± 0.014 vs. 0.069 ± 0.020, *p* < 0.001).

**Figure 7:**
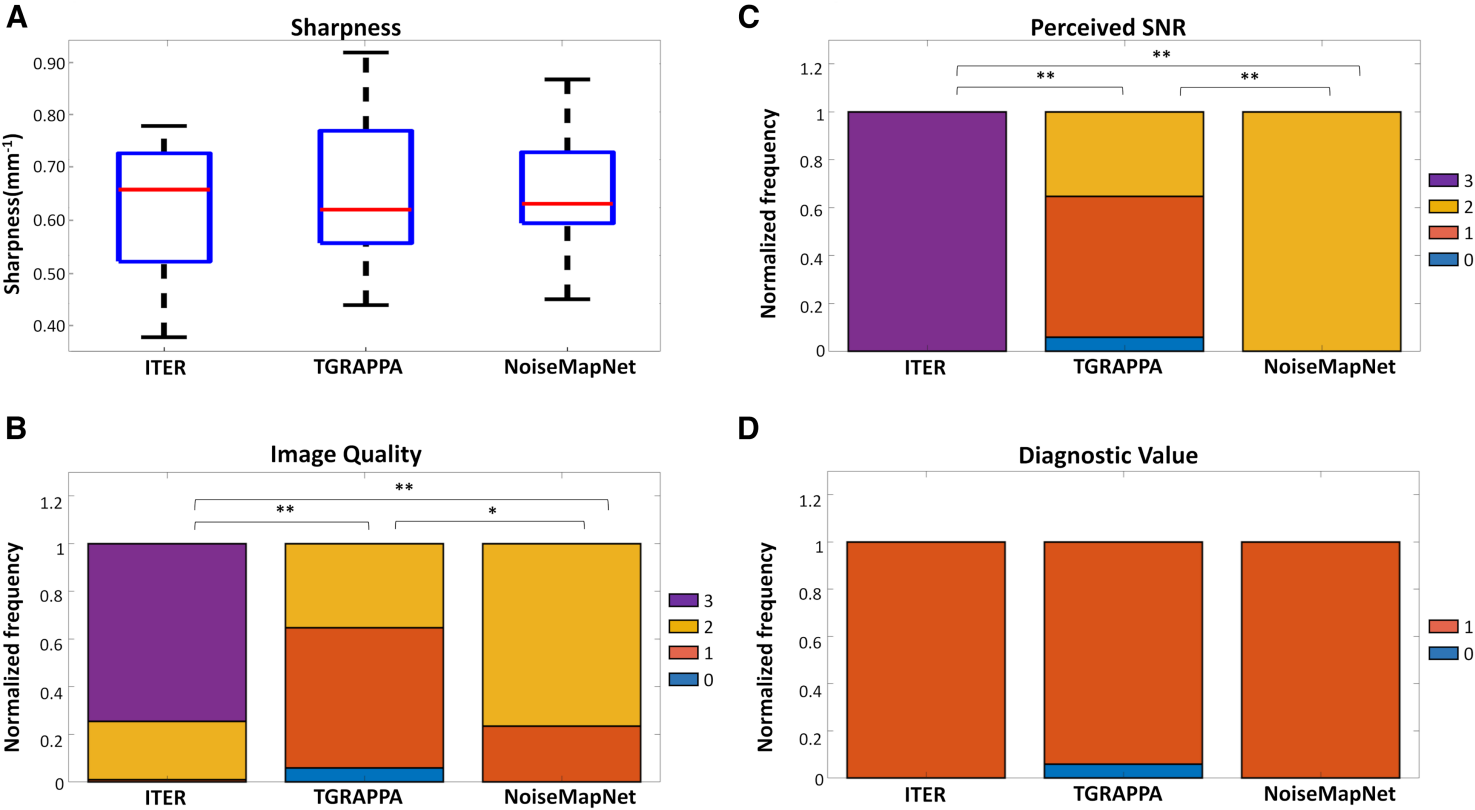
Quantitative and qualitative analysis of the three reconstruction techniques. (**A**) Image sharpness, (**B**) image quality, (**C**) perceived SNR, and (**D**) fraction of diagnostic myocardial segments are shown for all reconstruction techniques. Image quality was scored using a 4-point scale (0 = poor, 1 = major artifact, 2 = minor artifact, 3 = excellent). Perceived SNR (pSNR) was scored using also a 4-point scale (0 = very poor, 1 = major noise, 2 = minor noise, 3 = high SNR). Segment-wise diagnostic value (Yes/No) was assessed for each of the 16 American Heart Association myocardial segments. In the plots, ** represents *p* < 0.001 while * is *p* < 0.008.

Figure 7 shows the qualitative and quantitative analysis performed over all patients. There were no significant differences between all reconstructions in terms of myocardial sharpness: 0.63 ± 0.1 mm^−1^ (ITER) vs. 0.65 ± 0.1 mm^−1^ (NoiseMapNet) vs. 0.65 ± 0.1 mm^−1^ (TGRAPPA), *p* = 0.77. ITER led to higher pSNR (3.0 ± 0.0) than NoiseMapNet (2.0 ± 0.0, *p* < 0.001) and TGRAPPA (1.3 ± 0.6, *p* < 0.001). ITER also resulted in higher image quality (2.7 ± 0.4) than NoiseMapNet (1.8 ± 0.4, *p* < 0.001) and TGRAPPA (1.3 ± 0.6, *p* < 0.001). Importantly, NoiseMapNet yielded higher pSNR and image quality than TGRAPPA (*p* < 0.001 and *p* = 0.008, respectively). Finally, both ITER and NoiseMapNet led to 100% of the AHA segments being diagnostic, compared to 94% using TGRAPPA.

The reconstruction time of one entire dataset (6 slices, 80 dynamics) was 30 s for TGRAPPA with inline motion correction and 5 min for ITER, using a CPU. The computational cost of NoiseMapNet alone was 6 s using a GPU (2 min using a CPU) for one entire dataset. Therefore, the proposed NoiseMapNet-based reconstruction is feasible within 36 s.

## 4 Discussion

In this study, a fast and sharpness-preserving deep learning-based image denoising algorithm (NoiseMapNet) was developed and successfully combined with the standard TGRAPPA technique and a non-rigid image registration for the reconstruction of SMS bSSFP CMR perfusion images. NoiseMapNet provided higher image quality and pSNR than TGRAPPA alone, while preserving background signal and sharpness, and only requiring minimal additional computational time. Although ITER resulted in higher image quality and pSNR than the other two techniques, it was also associated with signal bias caused by the temporal regularization and longer reconstruction times.

In the scope of this work, the training of NoiseMapNet was performed using four chambers cine images which were acquired at different cardiac phases and had different contrast and geometry with respect to short axis perfusion images employed in this study. The rational of this choice was to demonstrate that the network is not specific to the image orientation used during the training process (as demonstrated in Supplementary Material) and can work for unseen image orientations. This is an important finding and suggests the strong potential of NoiseMapNet to be generalized to other CMR sequences and other clinical applications, which will be explored in future studies. However, the performance of NoiseMapNet was found to be related to the employed SNR and spatial resolution used during training (see Supplementary Material), which will need to be taken into account when applied to other applications. In this study, NoiseMapNet was trained using a unique level of noise, which was defined to approximate SNR found in typical images reconstructed using the employed SMS-bSSFP sequence with TGRAPPA. The simulated noise was also uniform in space. Integration of G-factor maps, or different noise levels during the training process may have the potential to improve the performance of NoiseMapNet.

Gaussian noise was used for the training of NoiseMapNet and this network was applied to magnitude images. Although noise in MR images often has a Rician distribution, it can be well approximated by a Gaussian distribution for moderate to high SNR. Based on this, deep learning-based image denoising using Gaussian noise has been previously used successfully in many MRI studies (41–43). Furthermore, post-processing algorithms can be used for the correction of the SNR-dependent intensity bias related to Rician or Non-central Chi distributions (44). However, this correction is expected to have minimal impact in the current context of myocardial perfusion where images are assessed visually for the presence of perfusion defect. Nevertheless, the extension of NoiseMapNet to model Rician and Non-central Chi noise distribution will be explored in the future, which may be relevant in the context of quantitative perfusion (23), where the improved temporal accuracy of NoiseMapNet with respect to ITER has potential to improve perfusion quantification.

NoiseMapNet was developed in this study for fast and efficient image-based denoising. Traditional techniques have been proposed for image denoising such as block-matching and 3D filtering (BM3D) (45), which has been used in a variety of applications. BM3D requires accurate assessment of noise levels to produce an optimal denoising. However, this is difficult to achieve as noise levels are patient-dependent (for example lower SNR are commonly observed in patients with large BMI, therefore requiring patient-specific tuning) and spatially varying due to g-factor maps. An underestimation of the noise level would result in imperfect noise removal, while an overestimated noise level will lead to increased blurring (46, 47). Conversely, NoiseMapNet was fully automated, did not require any user-defined parameters, and provided a uniform denoising across the entire field of view and across patients. Other deep learning architectures have been also successfully employed for image denoising, such as DnCNN (30). However, it was shown that U-Net provides better results at high noise levels and reduced training time compared to DnCNN (48). Nevertheless, the use of alternative networks (27), could represent an opportunity to improve upon the performance of NoiseMapNet, which will be the focus of future work. Incorporating perfusion images in the training with a semi-supervised or unsupervised deep learning approach may also have value in this context. Finally, we used the L2 norm as a loss function in NoiseMapNet. Although this loss is known to sometime cause slight blurring but still remain commonly used in image denoising (29, 30, 49), the use of alternative losses such as VGG-loss (50) or a combination of L2 norm with a VGG-loss (51) could be explored in the future.

NoiseMapNet was found to significantly improve image quality, pSNR, and diagnostic value over TGRAPPA, with minimal addition of reconstruction time (<6 s). These are very important findings as TGRAPPA is currently the main commercially available solution for the reconstruction of SMS images on the scanner. Therefore, the proposed solution shows high promises as an alternative online reconstruction of SMS perfusion images.

Regularization parameters for ITER were employed to trade-off image quality and temporal blurring, as previously optimized in (23, 38). Image reconstruction using ITER led to the highest image quality and pSNR but at the cost of a significantly longer reconstruction time than standard approaches and a loss of temporal fidelity of the perfusion signal, likely caused by its temporal regularization, as previously reported (21). ITER also has the potential to introduce temporal smoothing of transient artefacts, potentially leading to confounding factor of perfusion defect. Deep-learning techniques aiming at reducing the computation time of iteration reconstruction are usually trained on data from iterative reconstruction techniques and may also suffer from similar limitations. Conversely, the computational cost of NoiseMapNet is small. NoiseMapNet also enabled the accurate separation of background and noise signal, maintaining excellent temporal signal fidelity in the reconstructed images. This is particularly important as NoiseMapNet was shown to be superior on all aspects to TGRAPPA alone. Finally, unlike other deep-learning reconstruction or iterative reconstruction, the availability of estimated noise maps which can be used to confirm the absence of any anatomical structures, may play an important role to provide high confidence in the reconstructed NoiseMapNet images. Therefore, ITER and NoiseMapNet have distinct benefits and assessment of their individual diagnostic accuracy, which cannot be inferred from current image-based metric, is now needed to determine the value of each approach. Assessment of diagnostic accuracy will require a larger study in patients with coronary artery disease, ideally combined with FFR measurement for references.

Small temporal fluctuations were observed during the baseline and tail of the temporal profiles for both TGRAPPA and NoiseMapNet. Noise is not expected to be the cause of these fluctuations since the temporal profiles were measured as an average over a large region of interest. Instead, this may be caused by the presence of through-plane motion, which may be reduced for ITER due to the employed temporal regularization. Integration of prospective through-plane motion correction (52, 53) may help to reduce this effect.

NoiseMapNet may also have broader benefits. In MRI, the noise level is directly related to acquisition time and spatial resolution. Therefore, a robust image denoising approach has the potential to relax the current trade-off limits in MRI which may enable the acquisition of images with higher spatial resolution within the same scan time, similar spatial resolution with a shorter scan time, or a combination of both. NoiseMapNet could enable the use of higher acceleration factors which could be exploited to increase spatial coverage, spatial resolution or reduce the scan time of various MRI protocols.

This study has some limitations. First, a relatively small number of patients were included in this study. Second, SNR was not quantified in this study as it is challenging to measure in ITER images due to the iterative reconstruction which inherently thresholds and reduces noise inhomogeneously across the field of view (54). This is consistent with prior studies using compressed sensing, which have avoided reporting absolute SNR measurements but instead reported perceived SNR as performed in this study (54). Third, TGRAPPA was used as a reference for the temporal signal analysis. Although TGRAPPA has low SNR, the analysis was restrained to the first-pass of the contrast agent, thereby discarding low SNR baselines images. Furthermore, the signal was measured for each dynamic over a large ROI further minimizing the impact of noise. TGRAPPA does not use any temporal regularization and is therefore not expected to alter the temporal signal profile. Therefore, potential bias related to the use of TGRAPPA as reference signal for this analysis should have been kept to the minimum. Finally, although NoiseMapNet was shown to provide important benefits in terms of image quality, SNR, signal fidelity and computation time, its impact on diagnostic accuracy was not demonstrated. Therefore, a diagnostic accuracy study in a larger CAD patient cohort is now warranted to compare these techniques.

## 5 Conclusion

The proposed NoiseMapNet-based reconstruction enables fast, noise-filtered reconstruction of SMS perfusion images while preserving both sharpness and temporal signal profiles. This technique had superior performance than standard online TGRAPPA reconstruction. In comparison to ITER, NoiseMapNet resulted in lower image quality and pSNR but significantly shorter computation time suitable for inline use and no loss of temporal signal fidelity.

## Data Availability

The raw data supporting the conclusions of this article will be made available by the authors, without undue reservation.
