# Supplementary material for: Fast reconstruction of SMS bSSFP myocardial perfusion images using noise map estimation network (NoiseMapNet): a head-to-head comparison with parallel imaging and iterative reconstruction
Source: Front Cardiovasc Med. 2024 Jul 11;11:1350345. doi: 10.3389/fcvm.2024.1350345 (PMC11269255; doi:10.3389/fcvm.2024.1350345)
Supplement: Supplementary file 1 [file Datasheet1.pdf]

Supplementary Material

Fast reconstruction of SMS bSSFP myocardial perfusion images using Noise Map estimation network (NoiseMapNet): A head-to-head comparison with parallel imaging and iterative reconstruction

Naledi Lenah Adam[1], Grzegorz Kowalik[1], Andrew Tyler[1], Ronald Mooiweer[1,2], Alexander Paul Neofytou[1], Sarah McElroy[1,2], Karl Kunze[1,2], Peter Speier[3], Daniel Stäb[4], Radhouene Neji[1,2], Muhummad Sohaib Nazir[1,5], Reza Razavi[1], Amedeo Chiribiri[1], Sébastien Roujol[1*]

*** Correspondence:** Dr. Sébastien Roujol: sebastien.roujol@kcl.ac.uk

1 Supplementary Videos

1.1 **Supporting Information Video S1**: Video 1. Dynamic first pass perfusion images acquired for patient #1 using TGRAPPA, NoiseMapNet and ITER.

1.2 **Supporting Information Video S1**: Video 1. Dynamic first pass perfusion images acquired for patient #1 using TGRAPPA, NoiseMapNet and ITER.

1.3 **Supporting Information Video S2**: Video 2. Dynamic first pass perfusion images acquired for patient #2 using TGRAPPA, NoiseMapNet and ITER.

1.4 **Supporting Information Video S3**: Video 3. Dynamic first pass perfusion predicted noise maps acquired for patient #1 using NoiseMapNet.

Supporting Information Video S4: Video 4. Dynamic first pass perfusion predicted noise maps acquired for patient #2 using NoiseMapNet.

2 Supplementary Information

<u>**Hyperparameter importance and tuning**</u>

Hyperparameters of NoiseMapNet were initially optimized by visual inspection of loss functions and testing in one patient dataset. The importance of hyperparameters was assessed using a hyperparameter optimisation tool (Optuna) (1) which showed that the dropout rate selection had the highest influence (see Figure S1).

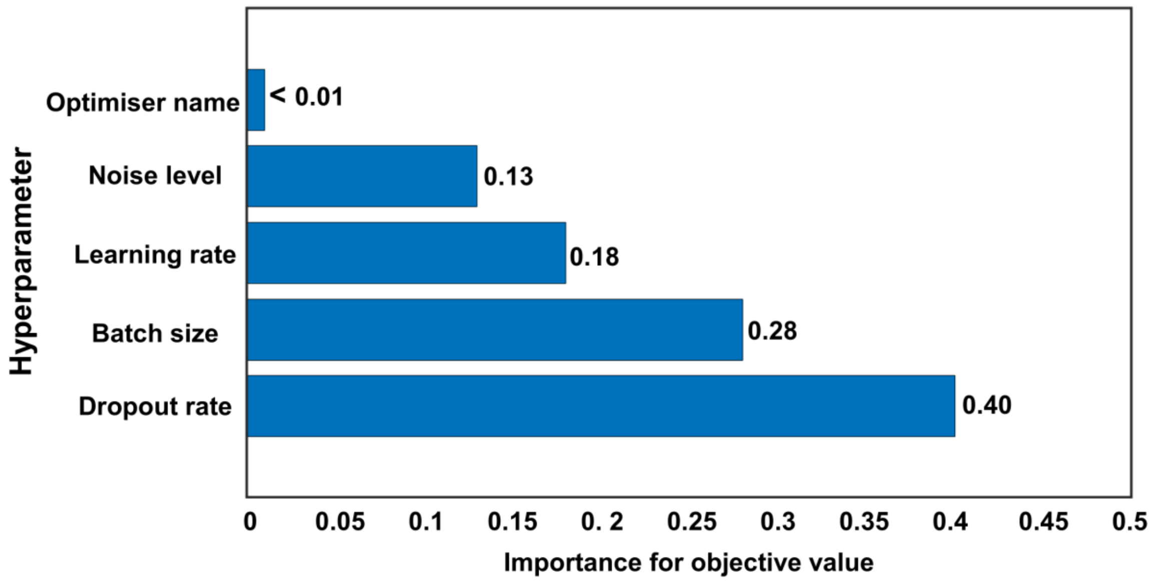

Figure S1. Hyperparameter importance plot comparing the influence of the dropout rate, batch size, learning rate, optimiser type and noise level.

[1]Akiba T, Sano S, Yanase T, Ohta T, Koyama M. Optuna: A next-generation hyperparameter optimization framework. InProceedings of the 25th ACM SIGKDD international conference on knowledge discovery & data mining 2019 Jul 25 (pp. 2623-2631).

A detailed analysis of the most impactful parameter selection (dropout rate) is presented in Figure S2. NoiseMapNet was trained using all optimized hyperparameters and a dropout rate of 0.1, 0.2, 0.3, 0.4, 0.6, 0.7. Training and validation losses are shown for these networks. To enable quantitative comparison of the networks on SMS perfusion images, the L1 norm of the noise map temporal average (L1-NMTA) was computed. This metric approximates the amount of true signal consistently removed by NoiseMapNet in a perfusion series, as a surrogate measure of accuracy in SMS perfusion images. A low L1-NMTA indicates minimal true signal removal through NoiseMapNet. L1-NMTA is reported for all networks.

The L1-NMTA plots demonstrate that a dropout rate <0.5 results in increased L1-NMTA (i.e. reduced image denoising performance). Increasing the dropout rate above 0.5 does not further improve the denoising process. Therefore, these results show that a dropout rate of 0.5 was optimal in the tested configuration given the denoising performance and training computational cost.

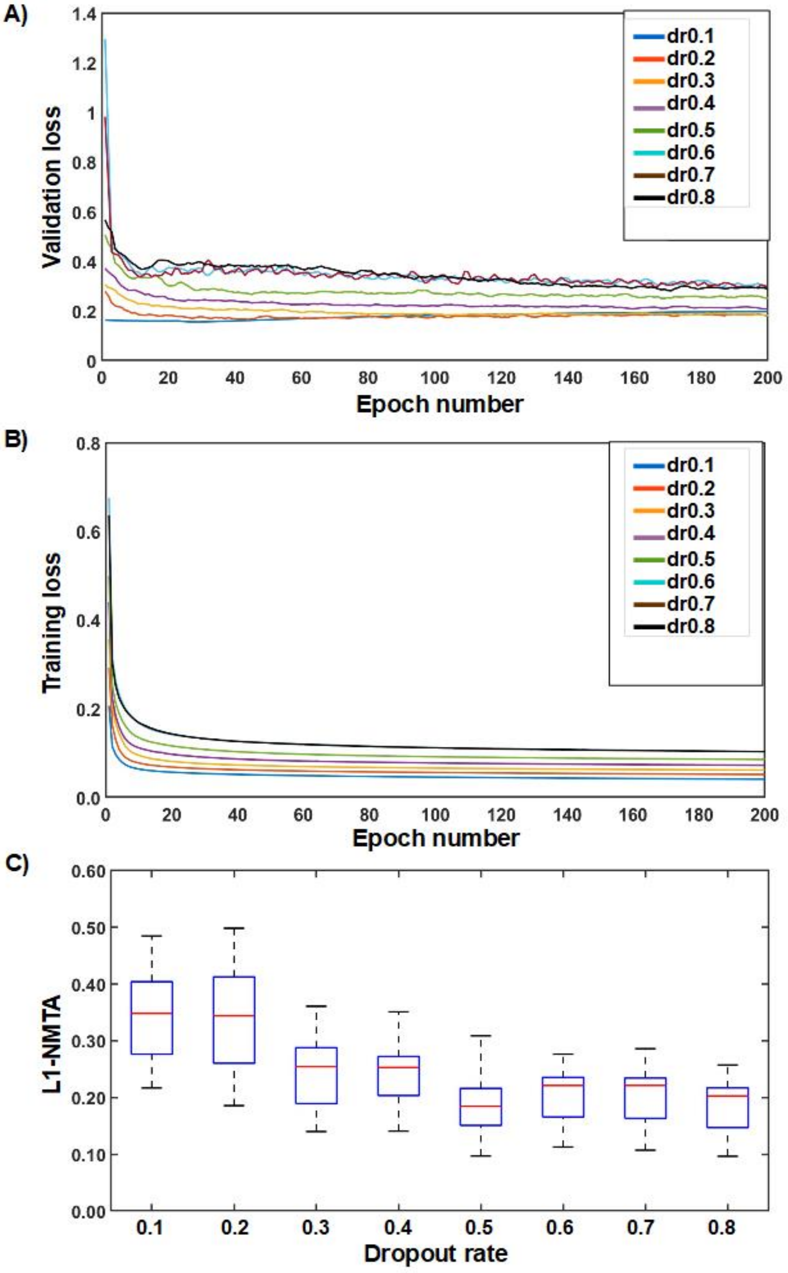

Figure S2. Training loss, validation loss and L1-NMTA for the different dropout rates. Based on L1-NMTA, a dropout rate of 0.5 was confirmed as optimal for the SMS-bSSFP perfusion datasets.

Impact of orientation, spatial resolution, and simulated noise level on NoiseMapNet

To investigate the impact of orientation on the training of NoiseMapNet, the network was trained using two different datasets acquired in short axis CINE images ($\text{NoiseMapNet}_{\text{SHAX}}$) and 4CH CINE

(NoiseMapNet$_{4CH}$, original NoiseMapNet). In addition, we investigated the effect of resolution on the training of NoiseMapNet. To this end, a third network was trained using 4CH CINE images (from the initial network) which were undersampled by a factor of 2 (NoiseMapNet$_{4CH_Undersampled}$). Finally, to study the impact of SNR, NoiseMapNet was also trained using three different levels of simulated noise, equivalent to a SNR of 5 (NoiseMapNet$_{4CH_SNR5}$), 10 (original) and 20 (NoiseMapNet$_{4CH_SNR20}$) in the left ventricular (LV) blood pool. The training loss and validation loss as well as denoised SMS perfusion images and corresponding L1-NMTA (as described in the previous section) are presented.

The impact of image orientation, spatial resolution, and simulated SNR on NoiseMapNet is shown in Figure S3 and S4. Training loss and validation loss plots cannot be compared directly since these were computed from different datasets, but confirm all networks were successfully trained. Similar visual image denoising performances were observed from the networks trained using four chamber and short axis images (Figure S4A). However, this example shows that the noise level used during the training process has a significant impact on the denoised images. A low noise level resulted in incomplete noise removal, while a high noise level resulted in increased blurring. There were no statistically significant differences in terms of L1-NMTA between NoiseMapNet$_{SHAX}$ and NoiseMapNet$_{4CH}$ ($p=0.55$) (Figure S4B). However, there was a significant difference in L1-NMTA between NoiseMapNet$_{4CH_Undersampled}$ and NoiseMapNet$_{SHAX}$ ($p<0.001$). The was also a significant difference between each pair of the three networks trained with different noise levels ($p<0.001$). These results therefore suggest that NoiseMapNet may be relatively invariant to image orientation but is dependent on simulated SNR and spatial resolution used during training.

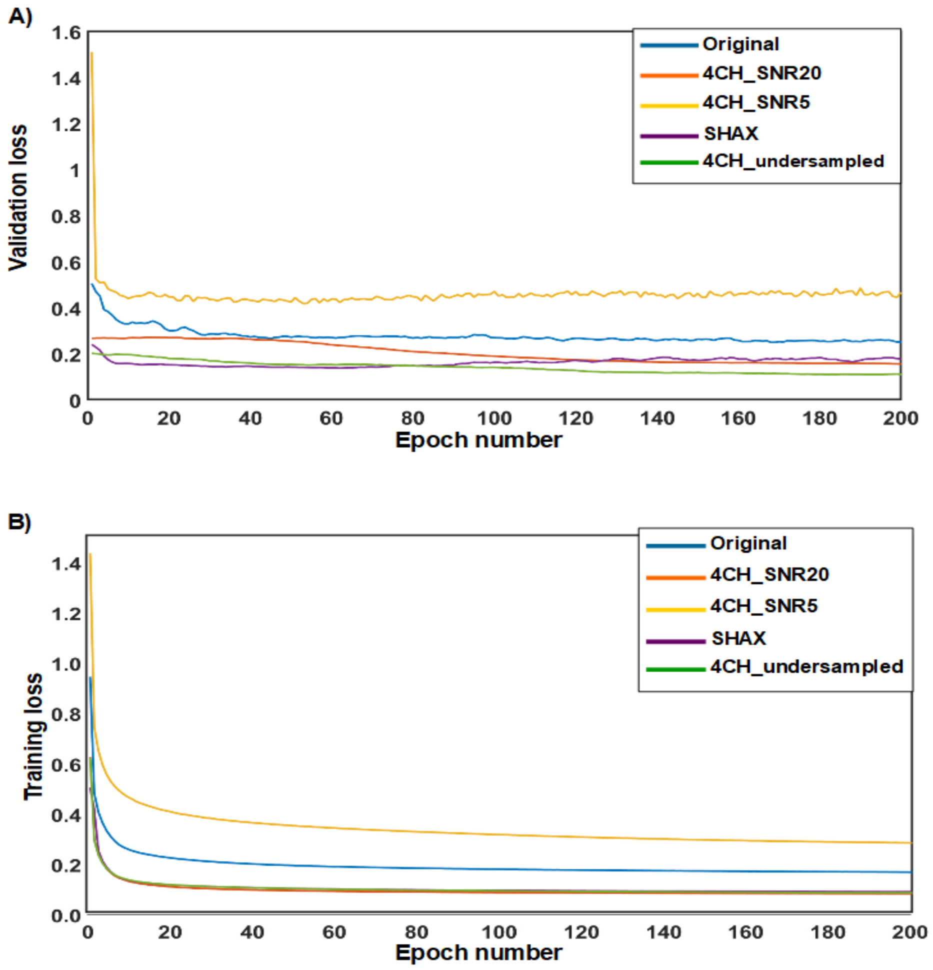

Figure S3. Impact of image orientation, spatial resolution, and simulated SNR on the training of NoiseMapNet. Training and validation loss plots are shown for NoiseMapNet trained with the original CINE four chamber dataset (4CH), a CINE dataset with different image orientation (short axis: SHAX), a CINE dataset with lower spatial resolution (original four chamber dataset undersampled by a factor of 2: (4CH_undersampled), and two four chamber CINE datasets using difference simulated noise level

(original four chamber dataset with doubled simulated noise level by a factor of 2: 4CH_SNR5, and original four chamber dataset with reduced simulated noise level by a factor of 2: 4CH_SNR20).

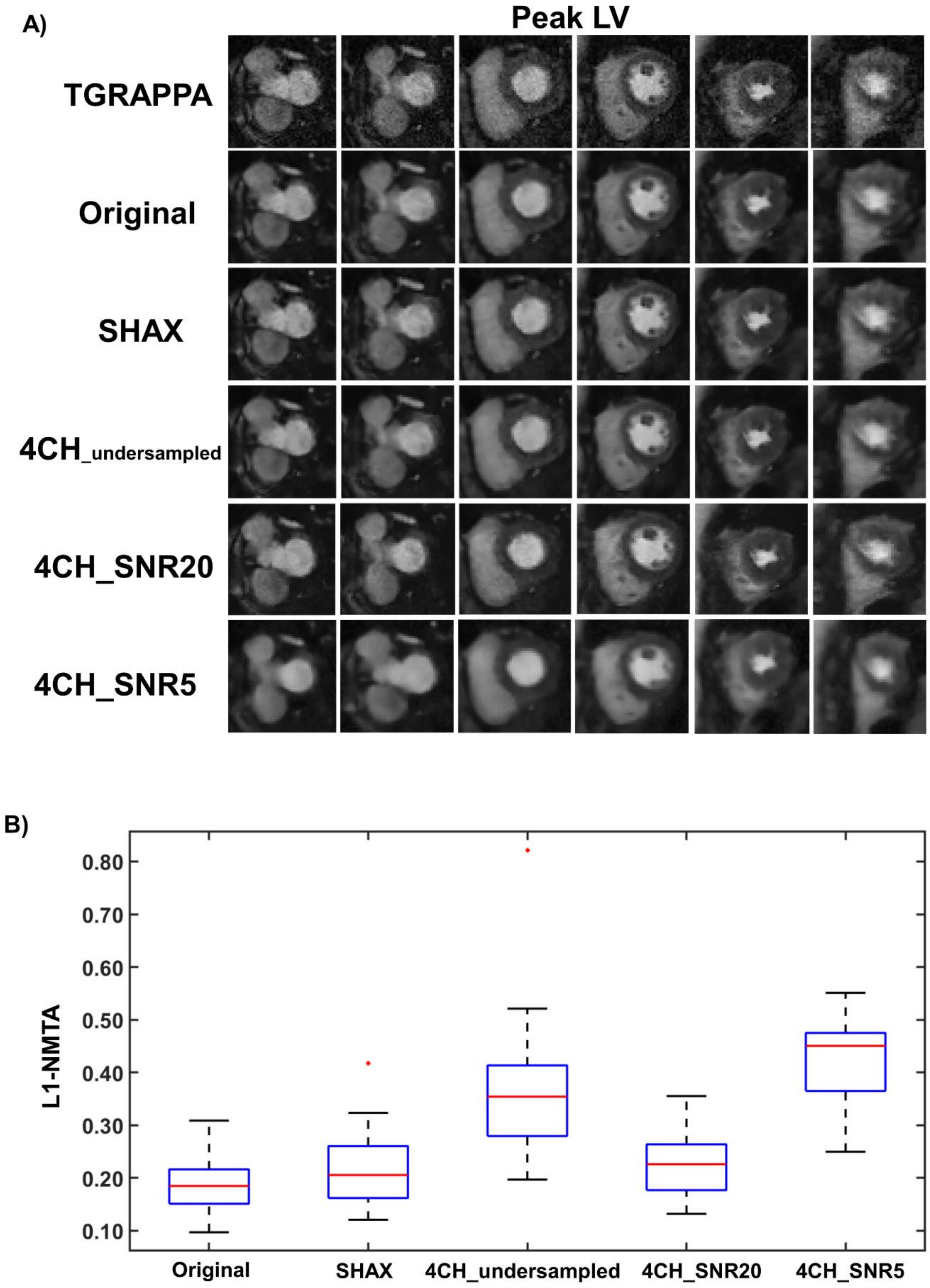

Figure S4. Impact of image orientation, spatial resolution, and simulated SNR on NoiseMapNet-based denoising of SMS perfusion images. Denoised SMS perfusion images and corresponding L1-NMTA are shown for NoiseMapNet trained with the original CINE four chamber dataset (4CH), a CINE dataset with different image orientation (short axis: SHAX), a CINE dataset with lower spatial resolution (original four chamber dataset undersampled by a factor of 2: (4CH_undersampled), and two four chamber CINE datasets using difference simulated noise level (original four chamber dataset with doubled simulated noise level by a factor of 2: 4CH_SNR5, and original four chamber dataset with reduced simulated noise level by a factor of 2: 4CH_SNR20).

References

1. Akiba T, Sano S, Yanase T, Ohta T, Koyama M. Optuna: A next-generation hyperparameter optimization framework. Proceedings of the 25th ACM SIGKDD international conference on knowledge discovery & data mining; 2019. p. 2623-2631.
